# Home-based cognitive telerehabilitation in multiple sclerosis: differential cognitive trajectories according to baseline impairment severity

**DOI:** 10.3389/fnbeh.2026.1894935

**Published:** 2026-08-31

**Authors:** Gianna Carla Riccitelli, Rosaria Sacco, Francesca Beeching, Giulia Mallucci, Giulio Disanto, Claudio Gobbi, Chiara Zecca

**Affiliations:** 1 Non-invasive Brain Stimulation Research Unit, Institute of Clinical Neuroscience of Southern Switzerland, Ente Ospedaliero Cantonale (EOC), Lugano, Switzerland; 2 Faculty of Biomedical Sciences, University of Southern Switzerland, Lugano, Switzerland; 3 Multiple Sclerosis Center, Institute of Clinical Neuroscience of Southern Switzerland, Ente Ospedaliero Cantonale (EOC), Lugano, Switzerland

**Keywords:** cognitive impairment, cognitive rehabilitation, multiple sclerosis, neurocognition, quality of life, telemedicine, telerehabilitation

## Abstract

**Background:**

Although cognitive impairment (CI) is common in multiple sclerosis (MS), conventional cognitive rehabilitation is often limited by accessibility and adherence challenges. Telerehabilitation offers an accessible alternative, but its efficacy across different CI severity levels remains unclear. The present prospective, single-center, single-arm interventional study with repeated measures aimed to identify changes in cognitive and psychosocial outcomes following a home-based cognitive telerehabilitation program with weekly synchronous coaching in MS patients with mild versus moderate CI, and to explore whether baseline impairment severity was associated with differences in change over time.

**Methods:**

Sixty-six MS patients (34 mild CI; 32 moderate CI) completed a 12-week home-based cognitive telerehabilitation program (five sessions/week), supported by weekly Skype coaching. Outcomes were assessed at baseline (T0), post-intervention (T1), and 3-month follow-up (T2). Cognitive and emotional-behavioral functioning were investigated using standardized, validated instruments in the MS population. Changes over time and between-group differences were analyzed using linear models.

**Results:**

At T1, both groups improved in attention, verbal memory, and subjective cognitive perception (all *p* < 0.05). Processing speed improved only in mild CI (*p* < 0.001), whereas executive function improved only in moderate CI (*p* = 0.007). At T2, mild CI further improved in attention and verbal memory (*p* = 0.003–0.035), while moderate CI improved only in processing speed (*p* = 0.006). Emotional-behavioral outcomes improved in both groups at T1 (anxiety *p* = 0.002; depression *p* = 0.023; fatigue *p* < 0.001) and were largely maintained at T2, with no statistically significant between group differences. Mental quality of life improved over time, whereas physical quality of life remained unchanged.

**Conclusion:**

Following the intervention, both groups showed improvements in cognitive and emotional-behavioral outcomes, with larger and more sustained changes in mild CI. These findings should be confirmed in randomized controlled studies.

## 1 Introduction

Multiple sclerosis (MS) is a critical immune-mediated demyelinating disease of the central nervous system associated with both physical and cognitive disabilities.

Cognitive impairment (CI) affects around 40 to 70% of MS patients independently of the disease stages and motor disability (Sharbafshaaer et al., 2022; Gaspari et al., 2023; Tacchino et al., 2023). It often involves deficits in memory, executive functioning, attention, and information processing speed, which significantly impair patients’ quality of life (QoL), undermining their social activities and employment (Maggio et al., 2024; Petracca et al., 2024).

Cognitive rehabilitation has emerged as a non-pharmacological approach to mitigate CI in the MS population, with studies showing improvements in memory, attention, and executive function after targeted interventions (Sharbafshaaer et al., 2022; Gaspari et al., 2023; Tacchino et al., 2023). Recently, technology-assisted cognitive rehabilitation approaches have been developed to overcome barriers related to accessibility and standardization (Tacchino et al., 2023; Özden et al., 2025).

Telerehabilitation is the remote delivery of rehabilitation services using telecommunication technologies, and it has been increasingly recognized as an effective and accessible alternative (Gopal et al., 2022; Petracca et al., 2024). Over traditional rehabilitation it has shown promising results, particularly when delivered synchronously and home-based, with notable benefits for cognitive, physical, and psychosocial outcomes. Additionally, it offers advantages in terms of accessibility and patient adherence, making it a promising alternative or complement to in-person rehabilitation (Sarpourian et al., 2024).

Recent evidence suggests that cognitive rehabilitation may positively influence not only cognitive performance but also, fatigue, mood, and quality of life in people with MS (Jeong et al., 2021; Petracca et al., 2024; Karakas et al., 2025) highlighting the importance of evaluating psychosocial outcomes alongside cognitive measures in MS rehabilitation (L’Abbate et al., 2025).

However, there is still limited information on whether patients with differing levels of baseline cognitive impairment respond equally to telerehabilitation. Establishing these differential effects is crucial, as it could significantly impact the tailoring of rehabilitation strategies to individual patient needs and the optimization of resource allocation.

In this study, we aimed to characterize changes in cognitive and emotional-behavioral outcomes following a home-based cognitive telerehabilitation program in people with MS. A secondary aim was to explore whether baseline cognitive impairment severity (mild vs. moderate) was associated with differences in the magnitude and durability of change across cognitive and psychosocial domains.

Given the absence of a control group, this study was not designed to establish causal effects but to provide exploratory evidence on change trajectories and subgroup differences.

## 2 Materials and methods

This was a prospective, single-center, single-arm interventional study with repeated measures conducted in people with MS.

### 2.1 Participants

Consecutive MS patients were recruited for the study at the Institute of Clinical Neuroscience of Southern Switzerland EOC, Lugano, Switzerland, between the 1st of September 2020 and the 30th of April 2024.

Inclusion criteria were: diagnosis of MS according to McDonald’s criteria (Thompson et al., 2018); age 18–70 years; clinical stability (defined as no relapses and no EDSS worsening) during the 3 months before enrolment; able to provide informed consent to study procedures; access to internet and home computer, and computer competency. Participants were excluded based on the following criteria: without impaired neuropsychological test scores (below the 5th percentile), dementia (MoCA ≤ 22) (Liew, 2019); relapse corticosteroid treatment within 3 months of testing; neurological/psychiatric disorders other than MS; alcohol/substance abuse; color-blindness; and motor or sensory defects that might interfere with the evaluation and training.

Of the 68 enrolled participants, two individuals in the moderate CI group discontinued the intervention because of conflicting schedules before the post-intervention assessment (T1). Consequently, no post-baseline outcome data was available for these participants.

All analyses were therefore performed on a per-protocol basis, including only participants who completed the intervention and all scheduled outcome assessments (*n* = 66).

**FIGURE 1:**
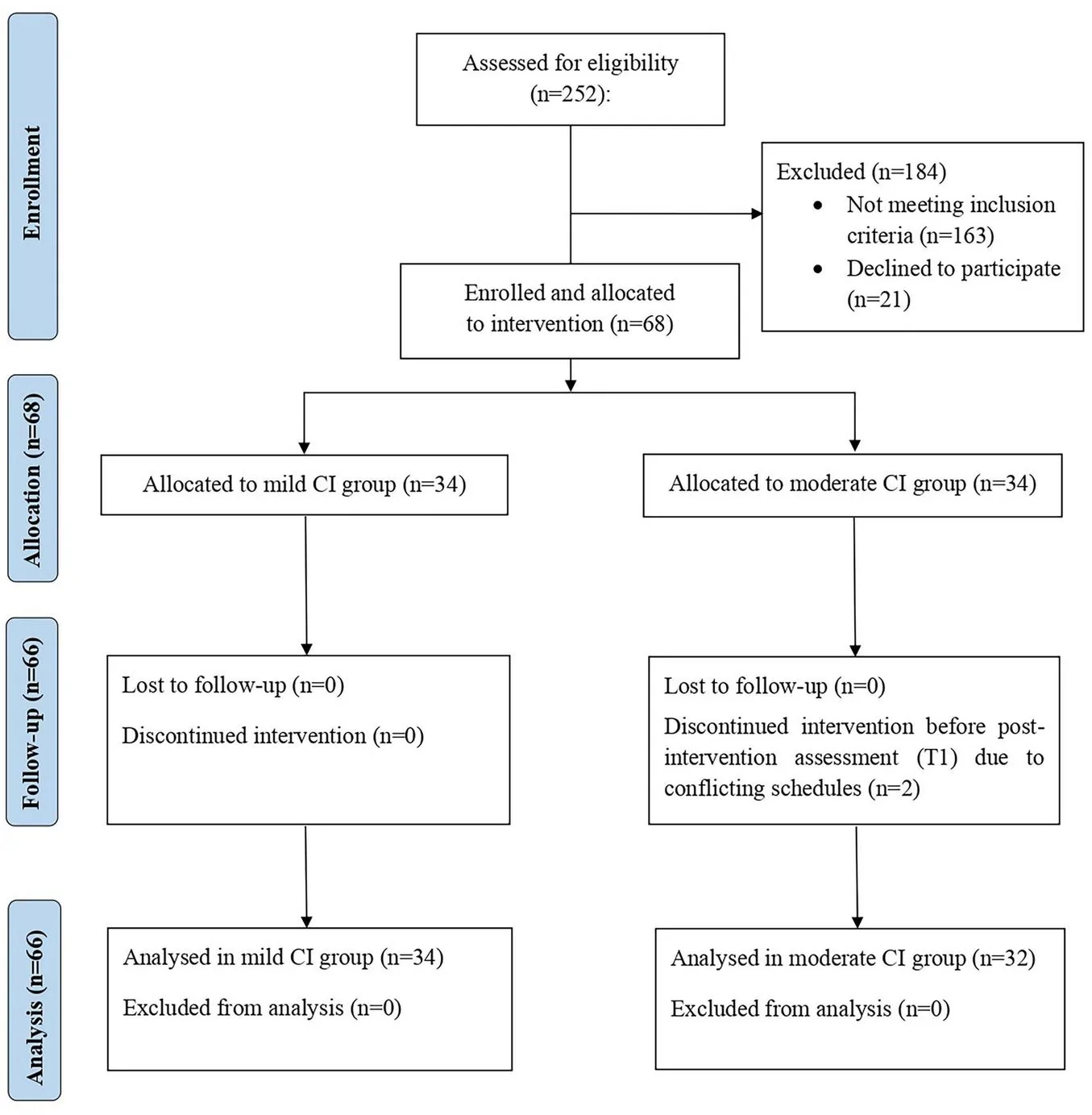
Participant flow chart. n, number, CI, cognitive impairment.

Additional information about eligibility, enrolment, and loss to follow-up is provided in Figure 1.

### 2.2 Sensitivity analysis

Because participant recruitment was based on consecutive enrollment, rather than an *a priori* sample size calculation, a retrospective sensitivity analysis using G*Power (v3.1) was conducted to evaluate the statistical power of the achieved sample size. Based on the effect size reported by Fuchs et al. (2019) (f = 0.28) (Fuchs et al., 2019), the final sample of 66 participants, assuming two groups, three repeated assessments (T0, T1, and T2), a correlation of 0.50 among repeated observations, and α = 0.05, provided approximately 80% power to detect effects of similar magnitude.

The study was approved by the Ethics Committee of Ticino (Comitato Etico Cantonale) on the 8th of September 2020. Ethical approval code: CE 3701. All participants gave informed consent.

### 2.3 Protocol of rehabilitation

The study lasted 6 months, during which patients underwent three visits lasting about 75 min each and 12 weekly Skype video calls of about 30 min each.

**FIGURE 2:**
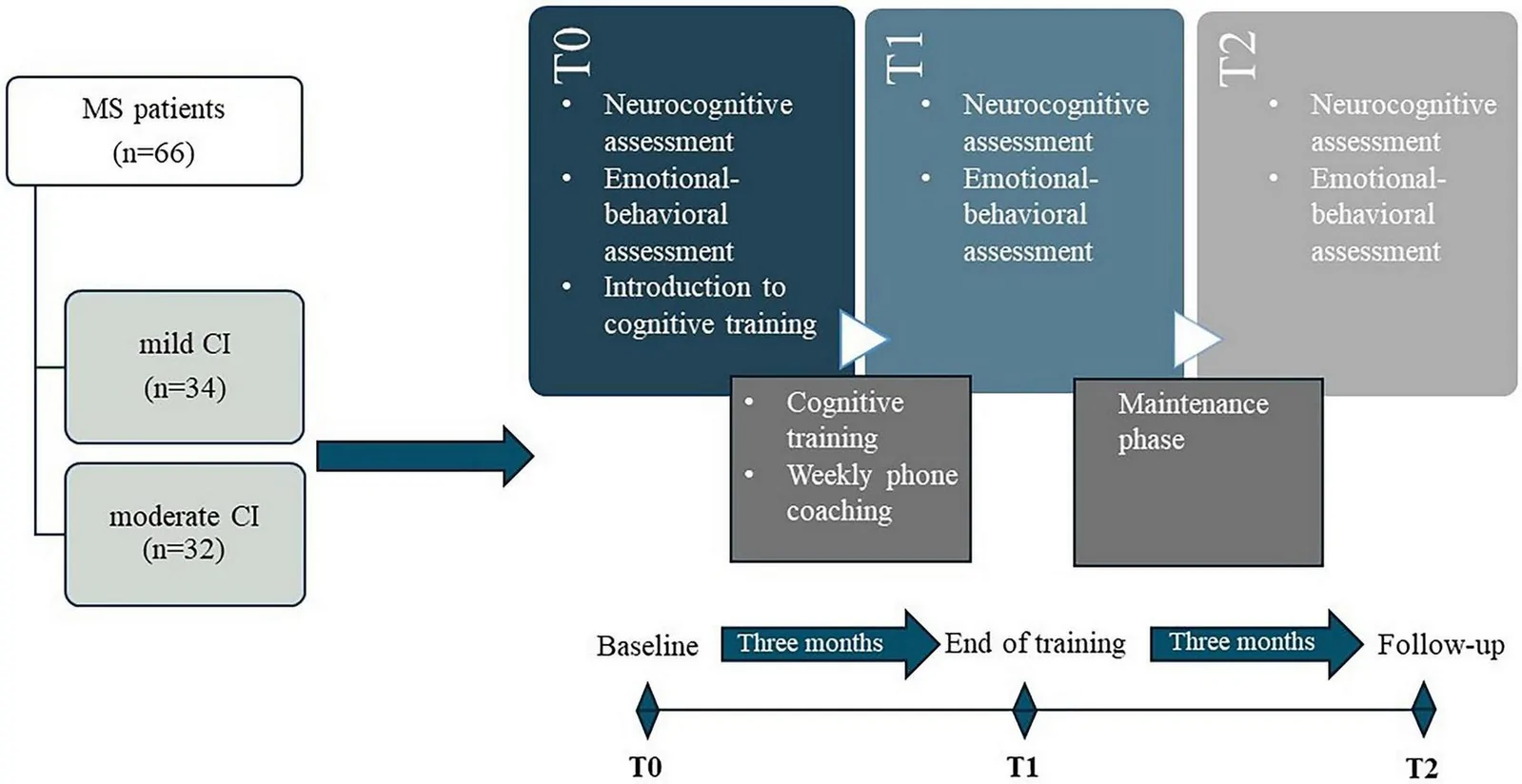
Study timeline. MS, multiple sclerosis; n, number; CI, cognitive impairment; T0, baseline; T1, end of three-month-training; T2, three-month follow-up.

Cognitive training lasted 12 weeks, 5 days a week. Each scheduled daily training session lasted approximately 60 min. Follow-up occurred three months after the end of the training period. Figure 2 provides a schematic timeline.

### 2.4 Cognitive assessment

Cognitive and emotional-behavior assessments were carried out by an experienced neuropsychologist before cognitive training (baseline; T0), at the end of training (after three months; T1) and at follow-up (three months after the end of training; T2) using two alternative versions (A at T0, B at T1, and A at T2) of the Brief Repeatable Battery of Neuropsychological Tests (BRB-N) (Rao et al., 1991). The A-B-A scheme was chosen because only two validated Italian BRB-N versions were available for longitudinal assessment (Amato et al., 2006; Goretti et al., 2014). Both were designed to minimize practice effects and to include normative corrections for reliable comparisons. Although normative retest data is available for the Italian BRB-N, reliable change indices were not applied because the study was designed to evaluate group-level longitudinal changes rather than individual change scores.

This commonly used battery in MS population, includes verbal memory tests [selective reminding test long-term storage (SRTlts), selective reminding test consistent long-term retrieval (SRTcltr), selective reminding test delayed retrieval (SRTd)]; visuo-spatial memory [10/36 spatial recall test (SPART), 10/36 spatial recall test delayed retrieval (SPARTd)]; information processing speed [symbol digit modality test (SDMT)] and attention (paced auditory serial attention test 3 (PASAT3) and 2 (PASAT2) seconds); and verbal fluency [word list generation (WLG)].

In addition, the Wisconsin Card Sorting Test (WCST) (Heaton and Staff, 1993) was used to investigate higher-order executive abilities.

BRB-N scores were normalized for age, sex, and education, according to Italian normative values (Amato et al., 2006; Amato et al., 2014), while the WCST global score was calculated according to Italian norms (Laiacona et al., 2000). Both BRB-N and WCST scores were transformed into z-scores and averaged to create a total composite z-scores for each cognitive domain (Sepulcre et al., 2006; Rocca et al., 2014).

At baseline, cognitive tests below the fifth percentile of controls were considered altered (Amato et al., 2006).

According to established criteria in MS literature (Curti et al., 2018), participants with one impaired test were classified as having mild CI, whereas those with two or more impaired tests were classified as having moderate CI. This classification was selected because it is widely adopted in the MS literature and facilitates comparison with previous cognitive rehabilitation studies using the BRB-N. It reflects clinically recognized cognitive phenotypes rather than a continuous measure of cognitive severity (Amato et al., 2006; Fischer et al., 2014; Curti et al., 2018).

The cognitive assessment was completed with the administration of the Multiple Sclerosis Neuropsychological Questionnaire (MSNQ) to investigate the subjective cognitive complaints of MS patients (Benedict et al., 2004).

Three emotional-behavioral scales were also administered to investigate: depression and anxiety symptoms with the Hospital Anxiety and Depression Scale (HADS) (Snaith, 2003), fatigue levels with the Modified Fatigue Impact Scale (MFIS) (Fisk et al., 1994) and patient QoL with the Multiple Sclerosis Quality of Life-54 (MSQoL-54) (Vickrey et al., 1995).

Neuropsychological assessments were administered by the same experienced neuropsychologist at baseline (T0), post-intervention (T1), and follow-up (T2).

Because of the longitudinal study design, the assessor was not blinded to the assessment timepoint. Outcome data was subsequently entered into the REDCap database by an independent researcher.

Moreover, because of the exploratory nature of this longitudinal study, no single primary outcome was prospectively prespecified. Cognitive performance represented the primary domain of interest, whereas emotional-behavioral functioning, fatigue, and quality-of-life measures were evaluated as secondary exploratory outcomes.

### 2.5 Intervention: cognitive training

All participants trained in the utilization of an online cognitive rehabilitation software (BrainHQ, Posit Science Corporation) completed the same exercise set.

The exercise set was selected to primarily target information processing speed, sustained and divided attention, and auditory/visual discrimination, which are among the most frequently cognitive domains affected in MS (DeLuca et al., 2020; Longinetti et al., 2024). The specific combination of exercises was not based on a previously published BrainHQ protocol using the identical exercise set, but was designed to emphasize processing-speed and attentional processes while also including tasks requiring executive control and functional cognitive efficiency.

This combination approach of information processing and attention training is consistent with previous trials adopting BrainHQ or CT of patients with MCI (Lin et al., 2020; Lee et al., 2024) and MS patients with CI (Vilou et al., 2020).

A more detailed description of the exercises selected for this study, their primary cognitive domains and their difficulty increase can be found in Supplementary Table 1.

Participants completed the training independently (asynchronous component), performing one session per day (∼45–60 min), 5 days per week for 12 weeks.

The BrainHQ platform adaptively adjusted task difficulty according to individual performance. Thus, when participants achieved higher accuracy or faster responses, task demands increased automatically; conversely, when performance declined, the software reduced or stabilized task difficulty. This adaptive structure allowed participants to train at an individualized level of challenge.

Weekly synchronous Skype sessions, approximately 30 min in duration were conducted by a blinded assistant researcher who was unaware of participants’ cognitive and psychosocial assessment results and cognitive impairment group. These sessions aimed to monitor adherence, address technical issues, and offer guidance and encouragement.

### 2.6 Statistical analyses

Statistical analyses were performed on the per-protocol population.

Analyses were conducted using IBM SPSS Statistics for Mac, Version 31 (IBM Corp., Armonk, NY, USA) (IBM Corp, 2025). Patient score trajectories were graphically depicted using R software, Version 4.5.1 (R Core Team, 2020).

The distribution of continuous variables was assessed using the Shapiro–Wilk test. Normally distributed variables are presented as mean ± standard deviation (SD) and compared using the independent-samples *t*-test, whereas non-normally distributed or ordinal variables are presented as median (interquartile range [IQR]) and compared using the Mann-Whitney U test.

Longitudinal changes were analyzed using linear mixed-effects models including effects for time, CI group, and the time × group interaction. Participant-specific random intercepts were included to account for within-subject correlations arising from repeated measurements. Linear mixed-effects models were chosen because they accommodate correlated longitudinal observations and incomplete repeated-measures data without requiring the sphericity assumption.

For cognitive outcomes, planned paired-sample *t*-tests were additionally performed within each group to explore short-term (T0 vs. T1) and medium-term (T1 vs. T2) changes attributable to the intervention.

Adherence was calculated as the percentage of prescribed training sessions completed, based on the number of sessions automatically recorded by the BrainHQ platform. It was described using the median, IQR, and range. Differences in adherence between groups were analyzed with the Mann-Whitney U test. Additionally, exploratory correlations between adherence and change in the main cognitive and psychosocial outcomes were evaluated using Spearman’s rank correlation coefficients.

All reported *p*-values are nominal (uncorrected). Because the analyses were exploratory and involved multiple correlated neuropsychological outcomes, no formal correction for multiple comparisons was applied.

This study was reported in accordance with the TREND (Transparent Reporting of Evaluations with Nonrandomized Designs) statement.

## 3 Results

### 3.1 Baseline characteristics

Based on predefined neuropsychological criteria, 66 participants were categorized as having mild CI (*n* = 34) and moderate CI (*n* = 32). Within the moderate CI group, 18 participants had two impaired cognitive tests and 14 had three impaired tests.

At baseline, 15 patients in the mild CI group (44.1%) and 18 patients in the moderate CI group (56.3%) had been receiving private psychotherapy for at least six months before starting cognitive training. No participants initiated private psychotherapy or other structured psychological support during the study period.

As showed in Table 1, the two groups did not differ significantly in sex distribution (*p* = 0.81), age (*p* = 0.74), education (*p* = 0.15), disease duration (*p* = 0.65), EDSS scores (*p* = 0.41), MS phenotype (*p* = 0.90), or treatment profiles, including DMTs (*p* = 0.26) and symptomatic therapy (*p* = 0.09).

**TABLE 1:** Demographic/clinical, neurological and neuropsychological characteristics of study participants.

Variables	MS patients	mild CI patients	moderate CI patients	*p*-value (mild CI vs. moderate CI)
	(*n* = 66)	(*n* = 34)	(*n* = 32)	
Sex (men/women) No.	26/40	14/20	12/20	0.81**
Mean age (SD) [year]	46.7 ( ± 10.6)	46.29 ( ± 10.63)	47.17 ( ± 10.69)	0.74*
Median education (IQR) [year]	13 (12–16)	12.50 (12–13.75)	12 (12–17)	0.15**
Mean disease duration (SD) [year]	12.9 ( ± 9.8)	13.41 ( ± 9.69)	12.29 ( ± 10.13)	0.65*
Median EDSS score (IQR)	2 (2–4)	2.5 (2–3.5)	3 (2–4)	0.41**
MS phenotypes (relapsing remitting /progressive) No.	52/14	27/7	25/7	0.90**
Collateral diagnosis (yes/no) No.	25/41	11/23	14/18	0.34**
DMT (none/ mAbs/ Oral/ Inject) No.	2/27/18/19	0/13/12/9	2/14/6/10	0.26**
Median symptomatic therapy (IQR)	1 (0–2)	1 (0–2)	1 (0–1)	0.09**
Mean z_verbal memory domain (SD)	−0.87 ( ± 1.0)	−0.43 ( ± 0.86)	−1.33 ( ± 0.97)	< 0.001*
Mean z_visual memory domain (SD)	−0.04 ( ± 0.90)	0.38 ( ± 0.69)	−0.49 ( ± 0.89)	< 0.001*
Mean z_attentional domain (SD)	−0.85 ( ± 0.67)	−0.62 ( ± 0.55)	−1.10 ( ± 0.71)	0.00*
Mean z_executive domain (SD)	−0.05 ( ± 0.67)	0.14 ( ± 0.57)	−0.26 ( ± 0.72)	0.02*
Median MSNQ (IQR)	28 (21–35)	29 (23.75–35)	30 (19.50–37.50)	0.69**
Median HADS-anxiety (IQR)	8 (5–11)	8 (6–10)	8 (5–15.5)	0.79**
Median HADS-depression (IQR)	5 (3–9)	5.5 (3–9)	5 (2–8)	0.69**
Median MFIS total score (IQR)	48 (37–56)	48 (37–57.5)	47 (37–55.5)	0.85**
Median MFIS cognition (IQR)	23 (19–27)	23 (18–27)	23 (19–26.25)	0.91**
Median MFIS physical (IQR)	21.5 (13–25)	22 (13–25.25)	20 (12–26)	0.89**
Median MFIS social (IQR)	4 (2–5)	4 (2–5)	3.5 (2–5)	0.92**
Mean MSQoL-54 mental (SD)	58.43 ( ± 19.48)	57.75 ( ± 18.15)	59.28 ( ± 21.37)	0.77*
Mean MSQol-54 physical (SD)	59.84 ( ± 16.29)	60.19 ( ± 15.53)	59.40 ( ± 17.51)	0.86*

MS, multiple sclerosis; CI, cognitive impairment; No., number; SD, standard deviation; IQR, interquartile range; EDSS, Expanded Disability Status Scale; DMT, Disease Modifying Therapy; mAbs, Monoclonal antibodies; MSNQ, multiple sclerosis neuropsychological screening questionnaire; HADS, Hospital Anxiety Depression Scale; MFIS, Modified Fatigue Impact Scale; MSQoL-54, multiple sclerosis quality of life-54. Data are presented as mean (SD) for normally distributed continuous variables and median (IQR) for non-normally distributed or ordinal variables.

*Independent-samples *t*-test;

**Mann–Whitney U test;

**χ^2^ test (or Fisher’s exact test, where appropriate).

Concerning cognitive measures, moderate CI patients exhibited significantly lower baseline z-scores across all major cognitive domains than those with mild CI, indicating a broader pattern of cognitive dysfunction rather than isolated impairment (all *p* < 0.001). It’s worth noting that subjective cognitive complaints did not differ between the two groups (MSNQ; *p* = 0.69).

Additionally, no significant group differences were found in emotional or behavioral measures, including: anxiety (HADS-A; *p* = 0.793), depression (HADS-D; *p* = 0.69), fatigue (MFIS total; *p* = 0.85), mental QoL (MSQoL-54; *p* = 0.77), and physical QoL (MSQoL-54; *p* = 0.86). See Table 1 for further details.

### 3.2 Cognitive outcomes over time

Overall adherence to the cognitive training program was a median of 80% (IQR, 70–90%; range, 60–100%) and did not differ significantly between the mild CI group (median, 80%; IQR, 70–90%; range, 60–100%) and the moderate CI group (median, 80%; IQR, 70–80%; range, 60–100%) (*p* = 0.50). Exploratory correlation analyses showed no significant associations between adherence and changes in the primary cognitive outcomes (PASA3, PASAT2, SDMT, and MSNQ) or psychosocial outcomes (MFIS and HADS-D) (all *p* > 0.05). No adverse events were reported during the study.

**FIGURE 3:**
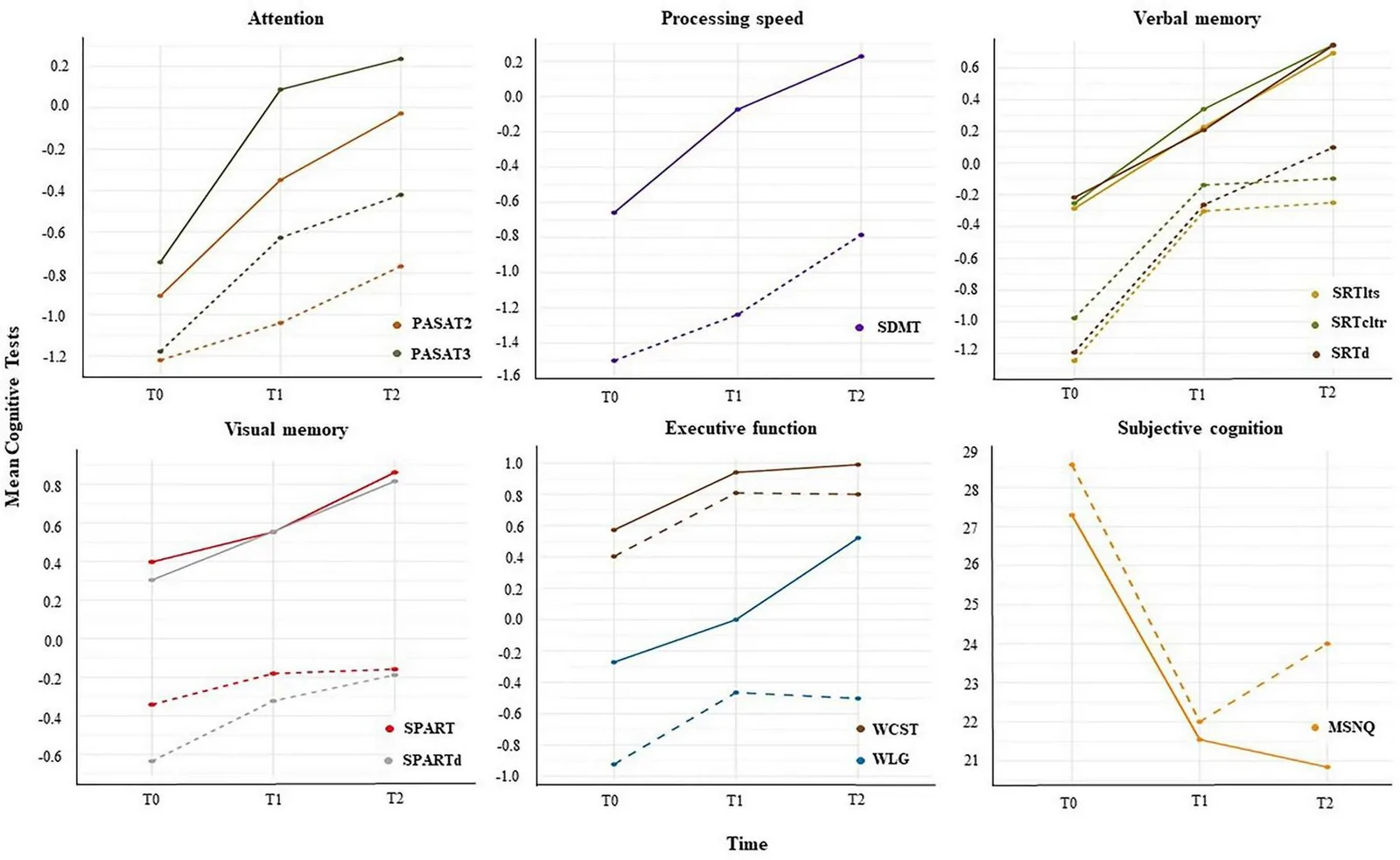
Cognitive scores of the mild CI group and the moderate CI group at baseline (T0), end of training (T1) and three-month follow-up (T2). Legend: Continuous line = mild CI; Dotted line = moderate CI. T0, baseline; T1, end of three-month-training; T2, three-month-follow-up; CI, cognitive impairment; PASAT2, Paced Auditory Serial Addition Test two seconds; PASAT3, Paced Auditory Serial Addition Test three seconds; SDMT, symbol digit modalities test; SRTcltr, selective reminding test consistent long-term retrieval; SRTlts, selective reminding test long term storage; SRTd, selective reminding test-delayed recall; SPART, spatial recall test; SPARTd, spatial recall test-delayed retrieval; WCST, Wisconsin Card Sorting Test; WLG, word list generation; MSNQ, multiple sclerosis neuropsychological screening questionnaire.

Significant improvements were observed over time in the cognitive tasks measuring sustained attention, information processing speed, and verbal memory. Figure 3 displays the cognitive performance of the mild CI group and the moderate CI group over time.

Performance on both PASAT and SDMT improved significantly over time. PASAT scores showed no significant time × group interactions, indicating comparable longitudinal changes between groups. While SDMT demonstrated a significant group effect (*p* = 0.004) and a trend toward a time × group interaction (*p* = 0.087), suggesting that improvements in processing speed may have differed according to baseline cognitive impairment.

For PASAT3, both time (*p* < 0.001) and group (*p* = 0.024) effects were significant. Short-term improvements (T0-T1) were observed in both mild CI (*p* < 0.001) and moderate CI (*p* = 0.004), and were maintained at follow-up. PASAT2 followed a similar pattern (time *p* < 0.001; group: *p* = 0.007), with both groups improving after treatment, while only the mild CI group showed additional improvement at follow up (*p* = 0.003).

SDMT scores also improved over time (*p* < 0.001), with a significant group effect (*p* = 0.004) and a trend toward interaction (*p* = 0.087). Specifically, the mild CI showed early improvement (T0-T1: *p* < 0.001), which was maintained at follow-up, whereas the moderate CI showed delayed improvement from T1 and T2 (*p* = 0.006).

In verbal memory, all SRT subtests (SRT-LTS, SRT-CLTR, SRT-D) showed significant time and group effects (*all p* < 0.05), with no interactions. Both groups improved at T1, with further improvement at T2 seen mainly in the mild CI group (e.g., SRT-LTS: *p* = 0.005; SRT-CLTR: *p* = 0.035). In contrast, moderate CI showed no long-term changes. For SRT-D, neither group demonstrated significant follow-up improvements.

Visuospatial memory, measured via SPART, showed limited changes. No significant time effects were found for SPART total scores (*p* = 0.359), though a group effect was detected (*p* = 0.003).

Neither group showed significant within-group improvements. SPART-D recall revealed a time effect (*p* = 0.033) and group effect (*p* < 0.001), but no meaningful within-group changes.

Verbal fluency (WLG) showed modest improvement, with significant time (*p* = 0.022) and group (*p* = 0.019) effects but no interaction. Within-group analyses were non-significant, though mild CI approached significance at T2 (*p* = 0.050).

Executive functioning, assessed by the WCST, improved over time (*p* = 0.002), with no group or interaction effects. Moderate CI showed significant short-term improvement (*p* = 0.007), which was maintained at T2, while mild CI showed no significant change.

Self-reported cognitive difficulties, as measured by the MSNQ, decreased significantly over time (*p* < 0.001), with no group or interaction effects. Both mild CI and moderate CI reported improvements at T1 (mild CI: *p* = 0.008; moderate CI: *p* < 0.001), which remained stable at T2.

Complete statistics are provided in Table 2.

**TABLE 2:** Cognitive effect of training over time and between mild CI and moderate CI groups.

Neurocognitive test	mild CI					moderate CI					Time	Time × group	Group
	T0	T1	T2	Paired T0 vs. T1	Paired T1 vs. T2	T0	T1	T2	Paired T0 vs. T1	Paired T1 vs. T2			
Mean PASAT3 z score (SD)	−0.74 ( ± 0.95)	0.09 ( ± 0.83)	0.24 ( ± 0.77)	**< 0.001**	0.915	−1.18 ( ± 1.16)	−0.63 ( ± 1.28)	−0.42 ( ± 1.30)	**0.004**	0.138	**< 0.001**	0.564	**0.024**
Mean PASAT2 z score (SD)	−0.90 ( ± 0.66)	−0.35 ( ± 0.71)	−0.03 ( ± 0.85)	**0.004**	**0.003**	−1.22 ( ± 0.77)	−1.04 ( ± 0.76)	−0.77 ( ± 1.01)	**0.023**	0.128	**< 0.001**	0.468	**0.007**
Mean SDMT z score (SD)	−0.65 ( ± 0.85)	−0.07 ( ± 1.19)	0.23 ( ± 1.18)	**< 0.001**	0.195	−1.48 ( ± 1.18)	−1.24 ( ± 1.13)	−0.79 ( ± 1.13)	0.164	**0.006**	**< 0.001**	0.087	**0.004**
Mean SRTlts z score (SD)	−0.39 ( ± 0.89)	0.01 ( ± 0.91)	0.54 ( ± 0.85)	**0.001**	**0.005**	−1.39 ( ± 1.12)	−0.46 ( ± 1.08)	−0.10 ( ± 0.99)	**0.001**	0.178	**< 0.001**	0.536	**0.016**
Mean SRTcltr z score (SD)	−0.46 ( ± 0.81)	0.03 ( ± 0.88)	0.49 ( ± 0.87)	**0.001**	**0.035**	−1.45 ( ± 1.02)	−0.50 ( ± 1.00)	−0.45 ( ± 1.27)	**< 0.001**	0.883	**< 0.001**	0.452	**0.003**
Mean SRTd z score (SD)	−0.44 ( ± 1.09)	0.14 ( ± 0.86)	0.55 ( ± 0.84)	**0.001**	0.060	−1.17 ( ± 1.11)	−0.34 ( ± 1.36)	−0.30 ( ± 0.97)	**0.046**	0.973	**< 0.001**	0.611	**0.015**
Mean SPART z score (SD)	0.40 ( ± 0.81)	0.55 ( ± 0.80)	0.86 ( ± 0.74)	0.542	0.051	−0.31 ( ± 1.39)	−0.18 ( ± 1.04)	−0.16 ( ± 1.12)	0.718	0.995	0.359	0.665	**0.003**
Mean SPARTd z score (SD)	0.36 ( ± 0.68)	0.56 ( ± 0.73)	0.82 ( ± 0.47)	0.467	0.102	−0.66 (0.76)	−0.32 ( ± 0.80)	−0.19 (0.81)	0.296	0.342	**0.033**	0.802	**< 0.001**
Mean WLG z score (SD)	−0.27 ( ± 0.87)	0.00 ( ± 1.08)	0.52 ( ± 0.85)	0.053	0.050	−0.91 ( ± 1.04)	−0.47 ( ± 1.23)	−0.50 ( ± 1.30)	0.315	0.762	**0.022**	0.197	**0.019**
Mean WCST z score (SD)	0.57 ( ± 0.81)	0.94 ( ± 0.17)	0.99 ( ± 0.17)	0.086	0.300	−0.41 ( ± 0.91)	0.81 ( ± 0.40)	0.80 ( ± 0.39)	**0.007**	0.862	**0.002**	0.416	0.121
Mean MSNQ score (SD)	27.20 ( ± 9.58)	21.55 ( ± 9.84)	20.84 ( ± 8.97)	**0.008**	0.254	28.64 ( ± 10.40)	22.00 ( ± 10.76)	24.00 ( ± 11.37)	**< 0.001**	0.574	**< 0.001**	0.320	0.560

CI, cognitive impairment; T0, baseline; T1, end of three-month-training; T2, three-month-follow-up; Z, z score; SD, standard deviation; PASAT3, Paced Auditory Serial Addition Test three seconds; PASAT2, Paced Auditory Serial Addition Test two seconds; SDMT, symbol digit modalities test; SRTlts-selective reminding test-long-term storage; SRTcltr - selective reminding test - consistent long-term retrieval; SRTd, selective reminding test-delayed recall; SPART, spatial recall test; SPARTd - spatial recall test-delayed recall; WLG, word list generation; WCST, Wisconsin Card Sorting Test; MSNQ, multiple sclerosis neuropsychological questionnaire. Reported *p*-values are nominal (uncorrected); results should be interpreted in the context of multiple comparisons. Bold text represents *p*-values ≤ 0.05.

### 3.3 Emotional and behavioral outcomes over time

Secondary exploratory outcomes related to emotional well-being, fatigue, and QoL are detailed in the Supplementary Table 2.

Across the total sample, significant time-related improvements were observed in emotional symptoms with reductions in both anxiety (HADS-A: *p* = 0.002) and depression (HADS-D: *p* = 0.023) scores. No statistically significant group or time × group interaction effects were detected.

Fatigue levels, assessed using MFIS, also improved significantly over time, both in total scores (*p* < 0.001) and within cognitive (*p* < 0.001), physical (*p* = 0.002), and social (*p* = 0.022) subdomains. No significant group or interaction effects were observed across any fatigue-related scores.

Regarding QoL, the mental health component of the MSQoL-54 showed a significant improvement over time (*p* < 0.001), while the physical component remained stable (*p* = 0.204). No significant group and interaction effects were found for either MSQoL-54 subscale.

## 4 Discussion

Following the intervention, improvements in cognitive and psychosocial outcomes were observed in MS patients with both mild and moderate CI. Improved performance was evident at the end of treatment (T1). It was largely maintained at 3-month follow-up (T2), although effect sizes varied by baseline impairment severity, with exploratory analysis suggesting larger cognitive improvements in patients with mild CI. In contrast, emotional-behavioral and quality of life outcomes improved over time similarly in both groups. Adherence was comparable between groups and was not significantly associated with outcome magnitude, suggesting that the observed subgroup differences were unlikely to be explained solely by differential training exposure.

Our findings are consistent with previous studies reporting improvements following cognitive rehabilitation in MS patients. In particular, Sharbafshaaer et al. (2022) demonstrated that an integrated home-based program significantly enhanced verbal learning, visuospatial memory, attention, and executive function, with sustained effects at 3 months (Sharbafshaaer et al., 2022). MS patients with mild CI showed greater improvement than those with moderate CI on domains requiring information processing speed, divided attention, and executive control. These findings are in line with prior evidence suggesting that preserved cognitive reserve and neural integrity may support greater responsiveness to cognitive rehabilitation. In Fuchs et al. (2019), for example, higher grey matter volume predicted larger gains in processing speed following restorative home-based training (Fuchs et al., 2019).

In contrast, moderate CI improved most in verbal learning and short-term visuospatial memory tasks, which may reflect the benefits of repeated practice and cueing strategies inherent to the telerehabilitation protocol. However, their progress plateaued earlier, and improvements in complex executive functions were smaller and less persistent over time, potentially reflecting greater difficulty in acquiring and maintaining cognitively demanding strategies.

These findings suggest that baseline cognitive status may be relevant when designing personalized telerehabilitation protocols. Mild CI patients may benefit from programs emphasizing speeded, multitasking challenges, whereas patients with more pronounced impairment may require slower-paced, repetitive, and ecologically orientated approaches to consolidate memory and attention improvements.

Our hypothesis aligns with Sharbafshaaer et al. (2022), who showed that an integrated home-based program improved multiple domains but highlighted the value of personalization to impairment level (Sharbafshaaer et al., 2022).

Both groups experienced reductions in fatigue, depression and anxiety, along with enhancements in mental QoL over time. These findings indicate an overall temporal change across the study population rather than evidence of differential psychosocial responses according to baseline cognitive impairment severity. Nevertheless, there are consistent with previous findings suggesting that cognitive rehabilitation can indirectly improve emotional well-being (Jeong et al., 2021; Petracca et al., 2024; Karakas et al., 2025). These findings need to be confirmed through larger, controlled studies.

The reduction in fatigue observed following the intervention may reflect improved efficiency of functional brain networks rather than changes in structural disease burden. Neurophysiological models suggest that MS-related fatigue arises from altered functional connectivity within networks responsible for cognitive control and effort regulation.

Repeated activation of these networks through cognitive training may reduce the neural effort required for everyday cognitive activities, thereby decreasing perceived fatigue.

More broadly, behavioral interventions appear to modulate fatigue across neurological conditions, supporting the concept that fatigue involves partially modifiable regulatory mechanisms (Bertoli and Tecchio, 2020; Tecchio et al., 2022; De Ventura et al., 2026).

The weekly synchronous coaching sessions may also have contributed to psychosocial improvements by providing interpersonal support, increasing motivation, reducing perceived isolation, and reinforcing self-efficacy. Therefore, improvements in mood, fatigue, and quality of life cannot be attributed exclusively to computerized cognitive training.

Neuroplasticity mechanisms may contribute to the observed changes, although this study did not directly assess underlying neural processes. As highlighted by Gaspari et al. (2023), task specificity and MS-tailored rehabilitation approach may improve treatment efficacy by aligning training demands with patients’ cognitive profiles (Gaspari et al., 2023). The adaptive nature of the software and the regular synchronous coaching sessions may have supported adherence and engagement, particularly among patients with greater impairment.

This study has several limitations. First, the absence of a control group represents the main methodological limitation of this study. Therefore, the observed changes cannot be attributed exclusively to the telerehabilitation intervention as spontaneous fluctuations in MS symptoms, regression to the mean, residual practice effects from repeated neuropsychological assessments, and nonspecific engagement or therapist-contact effects may also have contributed. This limitation is particularly relevant for emotional-behavioral outcomes and should be considered when interpreting the findings. Future randomized controlled studies are warranted to distinguish intervention-specific effects from these potential confounding factors.

Second, this was a single-center study conducted in a specialized neurorehabilitation setting, which may limit generalizability. Participants were likely more motivated, technologically equipped, and engaged than the broader MS population, potentially influencing adherence and observed changes.

Third, the absence of neuroimaging or objective biomarkers limits insight into the neural mechanisms underlying differential responses. Structural brain integrity, particularly grey matter volume, has been associated with rehabilitation outcomes and may contribute to the observed between-group differences.

Fourth, subgroup sample sizes may have limited the ability to detect smaller effects in secondary outcomes such as mood and fatigue. Larger, multicenter studies incorporating biomarkers, adaptive protocols, and extended follow-up are needed.

Fifth, although all participants completed the same exercise categories, the adaptive nature of the software may have resulted in different difficulty trajectories across individuals and cognitive severity groups. Progression data was monitored for adherence, but was not formally analyzed as an independent predictor of outcomes.

Finally, concurrent psychological support as a potential confounding factor. Several participants received private psychotherapy before enrolment and continued treatment throughout the study, while weekly Skype sessions provided additional interpersonal contact. Although these factors were similarly distributed between groups, their independent contribution to improvements in fatigue, mood and quality of life could not be quantified.

Because multiple correlated outcomes were analysed without adjustment for multiple comparisons, reported *p*-values should be considered nominal and interpreted as exploratory.

This study describes improvements in cognitive and emotional-behavioral outcomes following a home-based telerehabilitation in people with MS.

Although both severity groups experienced changes over time, those with milder impairment showed larger and longer-lasting improvements.

Although both severity groups showed changes over time, participants with milder CI tended to demonstrate larger and more sustained improvements across several cognitive outcomes. These findings should be interpreted cautiously, as statistically significant time × group interactions were generally not observed.

These findings highlight the potential relevance of baseline cognitive status in shaping rehabilitation responses and support the need for controlled studies to determine intervention-specific effects.

## Data Availability

The raw data supporting the conclusions of this article will be made available by the authors, without undue reservation.
