# Supplementary material for: Home-based cognitive telerehabilitation in multiple sclerosis: differential cognitive trajectories according to baseline impairment severity
Source: Front Behav Neurosci. 2026 Aug 31;20:1894935. doi: 10.3389/fnbeh.2026.1894935 (PMC13572147; doi:10.3389/fnbeh.2026.1894935)
Supplement: Supplementary file 1 [file Data_Sheet_1.docx]

Supplementary Material

| TITLE | PAGE |
| --- | --- |
| Supplementary Table 1 | 2-3 |
| Supplementary Table 2 | 4-6 |
| TREND Statement Checklist | 7-13 |

**Supplementary Table 1.** BrainHQ exercises.

| **Exercise** | **Module** | **Target domain** | **Description** |
| --- | --- | --- | --- |
| Hawk Eye | Brain Speed | Visual precision/ information processing speed | The user is required identify which bird is different from the others on the screen, as the exercise increases in difficulty with birds becoming more similar, backgrounds becoming more complex and the distance of stimuli from the screen center increases. The score is in milliseconds. As users improve, birds flash for fewer milliseconds. |
| Sound Sweeps | Brain Speed | Auditory processing speed | The user is required to listen to sounds that “sweep” up and down, and identify their direction, as the exercise increases in difficulty with varying sound frequencies and the time frame between two sweeps decreases. The score is in milliseconds. As users improve, sound sweeps speed up. |
| Visual Sweeps | Brain Speed | Visual processing speed | The user is required to watch two patterns that “sweep” in or out and identify their direction as exercise difficulty increases as the colors of the sweeps change, the directions of the sweep change and the thickness of the bars changes. The score is in milliseconds. As users improve, visual sweeps speed up. |
| Fine tuning | Brain Speed | Auditory perception and processing speed | The user is required to listen to a syllable and then determine which of the two presented in text on the screen they had just heard, as the exercise increases in difficulty by changing the pairs of syllables and the speaker’s voice from female to male. The user’s score is the level of similarity they reach. The higher the level, the more similar the two syllables sound. |
| Divided Attention | Attention | Divided attention | The user is required to watch two shapes as they flash on screen and decide whether they meet a certain criterion. The exercise changes by highlight different criteria from color to shape to interior. Furthermore, categories double up as exercise difficulty increases. As users improve, the images flash by more quickly. |
| Double Decision | Attention | Useful field of view; visual processing speed | The user is required to determine which car they saw at the center of the screen and locate where the Route 66 sign appeared in the periphery. Adding distractors, increasing the distance from the center, increasing the similarity between the cars and making the background more complex, increase exercise difficulty. The score is in milliseconds. As users improve, cars and road signs flash for fewer milliseconds. |
| Mixed signals | Attention | Higher attentive-executive abilities | The user is required to decide whether what they hear and see match in a particular way, with distractors becoming increasingly harder to ignore and less time to make a decision. |
| Relaxed and ready, now Freeze Frame | Attention | Alertness | The user is required to respond as images go by on screen and “freeze” when they see the target. The difficulty of the exercise increases as distractors get more similar, the stimulus category differs, and the target gets more subtle. As users improve, the target image appears less frequently. |
| Mind Bender | Intelligence | Cognitive flexibility | The user is required to choose the correct object based on which of two conditions the object pair falls under, as images and rules change and the time to answer decreases. The score is determined on how quickly users can respond accurately. |

**Supplementary Table 2.** Behavioral effects of training over time and between mild CI and moderate CI groups.

| **Behaviour scales** | | **Mild CI** | | | **Moderate CI** | | | **Time** | **Time * Group** | **Group** |
| --- | --- | --- | --- | --- | --- | --- | --- | --- | --- | --- |
|  |  | **T0** | **T1** | **T2** | **T0** | **T1** | **T2** | **p-value** | **p-value** | **p-value** |
| **HADS-A** | **Median (IQR)** | 8  (6-10) | 6  (4-10) | 6  (3-9) | 8  (5-16) | 6  (2-10) | 6  (3-9) | **0.002** | 0.391 | 0.891 |
|  | **Mean**  **(SD)** | 8.03  (±4.19) | 7.04  (±3.54) | 6.71  (±4.34) | 8.60  (±5.01) | 6.73  (±4.70) | 6.20  (±3.67) |  |  |  |
| **HADS-D** | **Median (IQR)** | 6  (3-9) | 6  (2-7) | 3  (2-7) | 5  (2-8) | 4  (1-6) | 3  (1-7) | **0.023** | 0.659 | 0.184 |
|  | **Mean**  **(SD)** | 6.24  (±3.81) | 5.20  (±3.08) | 4.52  (±3.46) | 5.37  (±3.66) | 3.77  (±2.65) | 3.30  (±3.52) |  |  |  |
| **MFIS Total** | **Median (IQR)** | 48  (37-58) | 33  (25-48) | 39  (26-51) | 47  (37-56) | 32.5  (13-49) | 34.5  (14-52) | **<0.001** | 0.890 | 0.371 |
|  | **Mean**  **(SD)** | 46.59  (±13.53) | 36.64  (±14.09) | 39.71  (±14.87) | 46.63  (±16.84) | 33.50  (±21.90) | 34.40  (±22.65) |  |  |  |
| **MFIS cognition** | **Median (IQR)** | 23  (18-27) | 16  (10-24) | 19  (12-25) | 23  (19-26) | 16  (6-22) | 18  (5-23) | **<0.001** | 0.983 | 0.384 |
|  | **Mean**  **(SD)** | 22.85  (±5.93) | 16.80  (±7.63) | 18.48  (±8.10) | 22.17  (±7.73) | 15.05  (±9.27) | 16.25  (±10.70) |  |  |  |
| **MFIS physical** | **Median (IQR)** | 22  (13-25) | 16  (12-23) | 18  (12-25) | 20  (12-26) | 14  (4-25) | 15  (6-26) | **0.002** | 0.994 | 0.377 |
|  | **Mean**  **(SD)** | 19.94  (±8.24) | 16.88  (±7.10) | 17.81  (±7.76) | 19.67  (±9.50) | 15.41  (±11.78) | 15.40  (±10.75) |  |  |  |
| **MFIS social** | **Median (IQR)** | 4  (2-5) | 3  (1.5-4) | 3  (2-5) | 3.5  (2-5) | 3.5  (0-5) | 3  (0-4) | **0.022** | 0.933 | 0.327 |
|  | **Mean**  **(SD)** | 3.74  (±1.86) | 3.20  (±1.94) | 3.43  (±1.86) | 3.73  (±2.32) | 3.00  (±2.56) | 2.75  (±2.30) |  |  |  |
| **MSQOL-54 Physical** | **Mean**  **(SD)** | 57.75  (±18.15) | 61.89  (±11.14) | 62.23  (±11.65) | 59.28  (±21.37) | 64.01  (±17.15) | 62.35  (±16.74) | 0.204 | 0.505 | 0.549 |
| **MSQOL-54**  **Mental** | **Mean**  **(SD)** | 60.19  (±15.53) | 65.28  (±17.14) | 67.67  (±15.35) | 59.4  (±17.51) | 66.42  (±18.35) | 66.93  (±21.82) | **<0.001** | 0.822 | 0.900 |

**Abbreviations**

CI, Cognitive Impairment; T0, baseline; T1, end of three-month-training; T2, three-month-follow-up; IQR, interquartile range; SD, Standard Deviation; HADS, Hospital Anxiety Depression Scale; MFIS, Modified Fatigue Impact Scale; MSQoL-54, Multiple Sclerosis Quality of Life-54. Effect significant at p ≤ 0.05

**TREND Statement Checklitst**

| **Paper Section/Topic** | **Item No.** | **Descriptor** | Reported? | |
| --- | --- | --- | --- | --- |
|  |  |  |  | Pg # |
| **TITLE and ABSTRACT** | | |  |  |
| Title and Abstract | 1 | - Information on how units were allocated to interventions | ✓ | 2 |
|  |  | - Structured abstract recommended | ✓ | 2 |
|  |  | - Information on target population or study sample | ✓ | 2 |
| **INTRODUCTION** | | |  |  |
| Background | 2 | - Scientific background and explanation of rationale | ✓ | 4-5 |
|  |  | - Theories used in designing behavioral interventions | N/A | - |
| **METHODS** | | |  |  |
| Participants | 3 | - Eligibility criteria for participants, including criteria at different levels in recruitment/sampling plan (e.g., cities, clinics, subjects) | ✓ | 5-6 |
|  |  | - Method of recruitment (e.g., referral, self-selection), including the sampling method if a systematic sampling plan was implemented | ✓ | 5-6 |
|  |  | - Recruitment setting | ✓ | 5 |
|  |  | - Settings and locations where the data were collected | ✓ | 5-6 |
| Interventions | 4 | - Details of the interventions intended for each study condition and how and when they were actually administered, specifically including: | ✓ | - |
|  |  | - Content: what was given? | ✓ | 8 |
|  |  | - Delivery method: how was the content given? | ✓ | 8 |
|  |  | - Unit of delivery: how were subjects grouped during delivery? | ✓ | 7-8 |
|  |  | - Deliverer: who delivered the intervention? | ✓ | 9 |
|  |  | - Setting: where was the intervention delivered? | ✓ | 5-6 |
|  |  | - Exposure quantity and duration: how many sessions or episodes or events were intended to be delivered? How long were they intended to last? | ✓ | 8 |
|  |  | - Time span: how long was it intended to take to deliver the intervention to each unit? | ✓ | 8 |
|  |  | - Activities to increase compliance or adherence (e.g., incentives) | ✓ | 9-10 |
| Objectives | 5 | - Specific objectives and hypotheses | ✓ | 5 |
| Outcomes | 6 | - Clearly defined primary and secondary outcome measures | ✓ | 7-8 |
|  |  | - Methods used to collect data and any methods used to enhance the quality of measurements | ✓ | 8 |
|  |  | - Information on validated instruments such as psychometric and biometric properties | ✓ | 7-8 |
| Sample size | 7 | - How sample size was determined and, when applicable, explanation of any interim analyses and stopping rules | ✓ | 6 |
| Assignment method | 8 | - Unit of assignment (the unit being assigned to study condition, e.g., individual, group, community) | ✓ | 6; Figure 1 |
|  |  | - Method used to assign units to study conditions, including details of any restriction (e.g., blocking, stratification, minimization) | N/A | - |
|  |  | - Inclusion of aspects employed to help minimize potential bias induced due to non-randomization (e.g., matching) | N/A | - |
| Blinding (masking) | 9 | - Whether or not participants, those administering the interventions, and those assessing the outcomes were blinded to study condition assignment; if so, statement regarding how the blinding was accomplished and how it was assessed | ✓  Partial blinding | 9 |
| Unit of Analysis | 10 | - Description of the smallest unit that is being analysed to assess intervention effects (e.g., individual, group, or community) | ✓ | 7-8 |
|  |  | - If the unit of analysis differs from the unit of assignment, the analytical method used to account for this (e.g., adjusting the standard error estimates by the design effect or using multilevel analysis) | N/A | - |
| Statistical methods | 11 | - Statistical methods used to compare study groups for primary methods outcome(s), including complex methods for correlated data | ✓ | 9 |
|  |  | - Statistical methods used for additional analyses, such as subgroup analyses and adjusted analysis | ✓ | 9 |
|  |  | - Methods for imputing missing data, if used | N/A | - |
|  |  | - Statistical software or programs used | ✓ | 9 |
| **RESULTS** | | |  |  |
| Participant flow | 12 | - Flow of participants through each stage of the study: enrollment, assignment, allocation and intervention exposure, follow-up, analysis (a diagram is strongly recommended) | ✓ | Figure 1 |
|  |  | - Enrolment: the numbers of participants screened for eligibility, found to be eligible or not eligible, declined to be enrolled, and enrolled in the study | ✓ | Figure 1 |
|  |  | - Assignment: the numbers of participants assigned to a study condition | ✓ | Figure 1 |
|  |  | - Allocation and intervention exposure: the number of participants assigned to each study condition and the number of participants who received each intervention | ✓ | Figure 1 |
|  |  | - Follow-up: the number of participants who completed the follow-up or did not complete the follow-up (i.e., lost to follow-up), by study condition | ✓ | Figure 1 |
|  |  | - Analysis: the number of participants included in or excluded from the main analysis, by study condition | ✓ | Figure 1 |
|  |  | - Description of protocol deviations from study as planned, along with reasons | N/A | - |
| Recruitment | 13 | - Dates defining the periods of recruitment and follow-up | ✓ | 5 |
| Baseline data | 14 | - Baseline demographic and clinical characteristics of participants in each study condition | ✓ | 9-10; Table 1 |
|  |  | - Baseline characteristics for each study condition relevant to specific disease prevention research | N/A | - |
|  |  | - Baseline comparisons of those lost to follow-up and those retained, overall and by study condition | N/A | - |
|  |  | - Comparison between study population at baseline and target population of interest | N/A | - |
| Baseline equivalence | 15 | - Data on study group equivalence at baseline and statistical methods used to control for baseline differences | ✓ | 9-10; Table 1 |
| Numbers analyzed | 16 | - Number of participants (denominator) included in each analysis for each study condition, particularly when the denominators change for different outcomes; statement of the results in absolute numbers when feasible | ✓ | 9; Table 2 |
|  |  | - Indication of whether the analysis strategy was “intention to treat” or, if not, description of how non-compliers were treated in the analyses | N/A | - |
| Outcomes and estimation | 17 | - For each primary and secondary outcome, a summary of results for each estimation study condition, and nominal p-values, estimated marginal means, and regression coefficients reported; standardized effect sizes and 95% confidence intervals were not calculated. | ✓ | 10-12; Table 2 |
|  |  | - Inclusion of null and negative findings | ✓ | 10-12 |
|  |  | - Inclusion of results from testing pre-specified causal pathways through which the intervention was intended to operate, if any | N/A | - |
| Ancillary analyses | 18 | - Summary of other analyses performed, including subgroup or restricted analyses, indicating which are pre-specified or exploratory | ✓ | 11-12 |
| Adverse events | 19 | - Summary of all important adverse events or unintended effects in each study condition (including summary measures, effect size estimates, and confidence intervals) | ✓  No adverse events reported |  |
| **DISCUSSION** | | |  |  |
| Interpretation | 20 | - Interpretation of the results, taking into account study hypotheses, sources of potential bias, imprecision of measures, multiplicative analyses, and other limitations or weaknesses of the study | ✓ | 12-15 |
|  |  | - Discussion of results taking into account the mechanism by which the intervention was intended to work (causal pathways) or alternative mechanisms or explanations | ✓ | 13-14 |
|  |  | - Discussion of the success of and barriers to implementing the intervention, fidelity of implementation | ✓ | 14 |
|  |  | - Discussion of research, programmatic, or policy implications | ✓ | 15 |
| Generalizability | 21 | - Generalizability (external validity) of the trial findings, taking into account the study population, the characteristics of the intervention, length of follow-up, incentives, compliance rates, specific sites/settings involved in the study, and other contextual issues | ✓ | 14-15 |
| Overall evidence | 22 | - General interpretation of the results in the context of current evidence and current theory | ✓ | 15 |

*From:*  Des Jarlais, D. C., Lyles, C., Crepaz, N., & the Trend Group (2004). Improving the reporting quality of nonrandomized evaluations of behavioral and public health interventions: The TREND statement. *American Journal of Public Health*, 94, 361-366. For more information, visit: <http://www.cdc.gov/trendstatement/>
